# Self-medication practice and associated factors among pregnant women in Addis Ababa, Ethiopia

**DOI:** 10.1186/s41182-018-0091-z

**Published:** 2018-05-01

**Authors:** Kidanemariam G/Michael Beyene, Solomon Worku Beza

**Affiliations:** 1 Ethiopian Food, Medicine and Healthcare Administration and Control Authority, Addis Ababa, Ethiopia; 2 GAMBY College of Medical Sciences, Addis Ababa, Ethiopia

**Keywords:** Self-medication practice, Pregnant women, Antenatal care, Health centers, Binary logistic regression, Addis Ababa, Ethiopia

## Abstract

**Background:**

Self-medication which is the act of obtaining and using one or more medicines without medical supervision is a common practice among pregnant women. Unless proper caution is taken, it may result in maternal and fetal adverse outcomes. In Ethiopia, information on self-medication practice during pregnancy is scanty. Hence, this study aimed to assess self-medication practice and associated factors among pregnant women in government health centers in Addis Ababa.

**Methods:**

An institution-based mixed study design using a sequential explanatory approach was employed among 617 pregnant women and nine key informants in Addis Ababa from May 8, 2017, to June 30, 2017. Multi-stage sampling technique was used to select study participants, and purposive sampling technique was used to select the key informants. The quantitative data were collected using a structured interview questionnaire and analyzed using Statistical Product and Service Solutions (SPSS) version 23.0 whereas semi-structured questionnaire was used for in-depth interviews. Binary logistic regression was used for quantitative data analysis, and thematic analysis method was used for qualitative data.

**Results:**

The prevalence of self-medication practice was 26.6%. Previous medication use (Adjusted odds ratio (AOR) = 4.20, 95% CI 2.70–6.53), gestational period (AOR = 0.63, 95% CI 0.41–0.98), education on self-medication (AOR = 0.36, 95% CI 0.21–0.62), previous pregnancy and delivery related problems (AOR = 1.71, 95% CI 1.06–2.76), and knowledge about risk of self-medication (AOR = 0.64, 95% CI 0.42–0.97) were significantly associated with self-medication practice. Lack of attention and priority of program designers, absence of strategies and guidelines; weak screening mechanisms, and regulatory enforcement were cited by the key informants as contributing factors for self-medication practices.

**Conclusions:**

Considerable proportion of pregnant women practiced self-medication, including medicines categorized to have high risks. Gestational period, previous medication use, education on self-medication, previous pregnancy- and delivery-related problems, and knowledge were significantly associated with self-medication practice. In addition, there are correctable gaps in program designing, screening of pregnant women, regulatory enforcement, and strategies and guidelines. Hence, necessary measures at all levels must be taken to reduce risks of self-medication during pregnancy.

## Background

Maternal and child health is a priority agenda of the global community [1, 2] and must be addressed using consistent and coordinated policies and programs [1, 3, 4]. Pregnancy is a period of great physiological changes to the mother and fetus [5]. During this period, a pregnant woman may take medication to alleviate pregnancy-related symptoms [6]. Medication use during pregnancy has been a concern both for the mother and fetus since the discovery of birth defects resulting from thalidomide crisis in early pregnancy in 1960s and teratogenic effects discovered related to use of diethylstilboestrol in 1971 [7–10].This necessitates critical evaluation of the risk level of medicines during pregnancy [11].

According to the United States Food and Drug Administration (USFDA) risk classification of medicines during pregnancy, medicines under category A show no risks in controlled studies and those medicines in category B have no evidence of human risk in controlled studies. Medicines in category C may have potential risks to the fetus. The risks cannot be ruled out but the medicines under category C should be used only if the potential benefits justify the potential risks to the fetus. Medicines in category D have positive evidence of human fetal risk, and those in category X are totally contraindicated in pregnancy because they have proven fetal risks [11].

Self-medication is defined as the act of using medications by patients or individuals to treat self-diagnosed disorders or symptoms on their own initiative [12, 13]. It is a behavior in which the individual attempts to solve his/her health problem without professional knowledge or advice [13]. An unauthorized purchase of medicines for self-medication use from nearby medicine retail outlets and other sources are increasing. It becomes the most common form of self-care [7, 14] though the irrational practices must be intervened and discouraged [15, 16].

When practiced properly, self-medication has a positive impact on individuals and health care systems. It needs health professional advices and access to appropriate information. In most developing countries in which the health system is inefficient and people access medicines easily from retail outlets, there may be an increased risk of self-medication during pregnancy [6].

Self-medication may cause serious structural and functional adverse effects on the fetus [17–19] including fetal toxicity, malformations, teratogen effects, and other potential harms [5, 8, 17, 20, 21]. Furthermore, it may cause low birth weight, premature birth, feeding problems; and respiratory problems in the fetus and affect the health of mother [8, 17, 21]. For many commonly used medicines, evidences of safe use in pregnant women have not been established. This is because medication safety information for pregnant women is limited due to the fact that pregnant women are often excluded from clinical trials of medicines [8, 22–24]. The limited medicine information has considerable contribution to maternal and neonatal mortality and morbidity, and fetal death [17, 25]. Despite this, studies showed that there is a high level of self-medication use among pregnant women [22, 24].

Globally, self-medication practice during pregnancy has been increasing and found to be high in many regions of the world, especially in developing countries [22, 26, 27]. The type, extent, and reasons for its practice however vary. Studies showed that both modern and herbal medicines are commonly used for self-medication in developing countries. This is due to easy access to medicines [6, 16, 28, 29]. Herbal medicine use poses potential risks to the mother and fetus due to the fact that the composition and safety parameters of these products are unknown [27].

There are different reasons for self-medication among pregnant women in different countries. It is associated with factors such as age, income, education level, knowledge, access to medicines, time [14, 16], perception towards risk of self-medication [26, 30–32], previous medication use, gestational age, and occupation [33, 34]. Studies found that self-medication practice is high during pregnancy in Europe (Western, Northern, and Eastern); North and South America and Australia (66.9%); Yazd, Iran (> 35%); Ahvaz, south Iran (30.6%); Hyderabad, Pakistan (37.9%); and Paraná, Brazil (94.67%) [34–37]. However, low prevalence rates of self-medication were reported in Peru (10.2%), Portugal (1.3%), the Netherlands (12.5%), Brazil (16.4%), and Arak city, Iran (12%) [18, 38–41].

In Africa, both modern and herbal medicines are commonly used for self-medication. Studies conducted in different parts of Nigeria revealed that 72.4% in Uyo, 31.5% in Ado-Ekiti, 63.8% in Ibadan, and 85% in Jos of pregnant women practiced self-medication [19, 29, 33, 42]. A study conducted in the Democratic Republic of Congo found that 59.9% of pregnant women used self-medication [6]. In Ethiopia, an institution-based study conducted in governmental health centers in Bahirdar showed that 25% of pregnant women reported self-medication [27]. A hospital-based study conducted among pregnant women in Jimma University specialized hospital showed that the prevalence of self-medication was 20.1% (16). A similar institution-based study conducted in hospitals in Mekelle showed 9.5 and 2% self-utilized modern and herbal medicines respectively [43].

Maternal and child health is one of the major priority agendas of the government of Ethiopia [44] even though there is a remarkable achievement observed in the reduction of under-five mortality rates. The reduction in mortality and morbidity in neonatal age groups however is not impressive. Prematurity is the most common cause of neonatal mortality [44]. Preventing pregnancy- and delivery-related problems associated with self-medication practice helps to protect the health of the mother and fetus [17, 25]. Identifying the extent and determinants of self-medication among pregnant women may contribute in achieving the targets of Sustainable Development Goal (SDG).

However, in Ethiopia despite the potential risks of self-medication, researches on the extent and determinants of self-medication during pregnancy are scanty. The findings of this study will provide information for policy makers and relevant stakeholders to develop strategies and appropriate interventions to prevent the risks associated with self-medication practice during pregnancy.

Hence, this study aimed to assess self-medication practice and associated factors among pregnant women attending antenatal care in government health centers in Addis Ababa, Ethiopia.

## Methods

### Study design and setting

An institution-based cross-sectional mixed study method was employed with a sequential explanatory design to triangulate the quantitative data with the qualitative data. Data were collected from May 08 to June 30, 2017, in Addis Ababa.

Addis Ababa is the diplomatic capital of the African Union and capital city of Ethiopia, located about 2500 m above sea level. It has ten sub-cities and 116 *woredas* [45]. The city has an estimated population of 3.2 million of which 52.6% are female [46]. During the study period, there were 92 functional government health centers, 378 pharmacies, 278 drug shops, and 777 different types of clinics in Addis Ababa [46].

### Study participants

The study participants were pregnant women attending antenatal care (ANC) in selected government health centers in Addis Ababa during the study period. The key informants for the in-depth interviews were directors, team leaders, ANC program coordinators, and ANC case team leaders in their current position.

### Sample size and sampling procedure

The sample size was determined by using the single population proportion formula for the prevalence of self-medication practice and double population proportion formula for predictor variables assuming 25% prevalence of self-medication practice during pregnancy from a study conducted in Bahirdar [27] at 95% confidence level, 5% marginal error and design effect of two. Anticipating 10% non-response rate, the final sample size was 634 pregnant women. The sample size for the qualitative study was guided by the degree of information saturation based on preliminary analysis during data collection. Accordingly, a total of nine key informants were purposively selected and interviewed in order to gather more explanatory opinions and deepen information complementing the quantitative findings.

Multi-stage sampling technique was employed to select the study participants. There were a total of 92 functional government health centers in Addis Ababa. The lists of the health centers were obtained from Addis Ababa Health Bureau. All health centers were listed, and table of random numbers was employed to randomly draw the required number of health centers. Eighteen health centers (20% of the total health centers) were selected using simple random sampling technique. Proportional numbers of pregnant women were assigned to each health center based on the flow of pregnant women per month taking one previous year antenatal care records. Then, systematic random sampling technique with fixed sampling interval was used to draw 634 pregnant women attending antenatal care in the selected health centers. For the qualitative study, purposive sampling technique was used. The key informants were from the Ministry of Health; Addis Ababa Health Bureau (AAHB); Ethiopian Food, Medicine and Healthcare Administration and Control Authority; and health centers.

### Data collection tools, procedures, and quality assurance

Quantitative data were collected using an interviewer-administered structured questionnaire. The structured interview questionnaire was adapted from previous studies [27, 29, 35, 47] and modified to fit the current study. The questionnaire was originally prepared in English, translated to Amharic language, and then back translated to English to validate consistency of meaning. Before the commencement of the actual data collection, the questionnaire was pretested on 5% randomly selected pregnant women attending antenatal care in government health centers who were later excluded from the study. Accordingly, the questionnaire was slightly modified.

The questionnaire consisted of questions related to socio-demographic factors, obstetric factors, previous medication use, education and counseling, and knowledge about risk of self-medication practices. To assess knowledge about risk of self-medication practice of the pregnant women, 12 questions in a five Likert scales (0–4 scale) were used and each question was coded and computed, and scores were dichotomized into good knowledge (Participants who scored ≥75% on knowledge based questions) and poor knowledge (Participants who scored < 75% on knowledge based questions). The collected data were checked for its consistency and completeness before any attempt to enter code and analyze it. Finally, Epi-Info version 7.2.1.0 was used to control and manage errors resulting from the data entry process.

One-on-one, face-to-face, in-depth interviews were carried out with key informants using semi-structured, open-ended interview questionnaire with flexible probing techniques to elaborate original responses of the interviewee. The interview questions were classified into two sections. The first section focused on personal and professional information such as age, professional background, work experience, and educational background. The second section was designed to explore the stakeholders view on self-medication practice, screening mechanisms, education on self-medication, regulatory enforcements, reasons for self-medication, and availability of strategies, guidelines and charts or forms.

The interviews were conducted by principal investigators. The interviews were audio recorded and notes were taken properly. The participants were interviewed at the time and location of their choice. The average duration of the interviews was approximately 35 min.

### Data management and analysis

The collected quantitative data were entered into Epi-Info version 7.2.1.0 and analyzed using SPSS version 23.0. Participants’ socio-demographic characteristics, obstetric factors, previous medication use, education and counseling, and knowledge about risk of self-medication were presented using relevant descriptive statistics. Univariate analysis was done at 25% level of significance to screen out potentially significant independent variables. Multivariable logistic regression was performed by using the relevant independent variables. The association between the dependent variable and independent variables were assessed using binary logistic regression. Hosmer and Lemeshow goodness-of-fit test was used to check the adequacy of the final model and the model fitted to the data well (*p* value = 0.434). Results were expressed as crudes, adjusted odds ratio, and 95% confidence interval. Variables with *p* value < 0.05 were considered as statistically significant.

For the qualitative study, the audio records and notes of the interviews were transcribed using a verbatim transcription technique. The transcribed scripts were intensively read, and the data were categorized into themes. Thematic analysis method was used to analyze the data. The analysis was facilitated using OpenCode version 4.0.2.3 software. To check the accuracy of the translation, one of the recordings was translated and transcribed by a bi-lingual expert and compared with the primary work. In reporting the findings, codes were used to maintain anonymity of the key informants. Furthermore, the findings of the study were communicated to five of the key informants for authenticity of the transcripts and interpretations.

## Results

### Socio-demographic characteristics

Out of the 634 study participants, 617 pregnant women participated in the study making the response rate of 97.3%. The majority of the respondents, 251 (40.7%), were in the age group of 25–29 years, and 199 (32.3%) participants had completed secondary school (grade 9–12). Two hundred and twenty nine (37.1%) of the respondents were housewives, and 273 (44.2%) had a monthly average family income of 3000–5999 ETB (Table 1).

**Table 1:** Socio-demographic characteristics of the study participants, Addis Ababa, 2017 (*n* = 617)

Variables		*n* (%)
Age in year	18–24	194 (31.4)
	25–29	251 (40.7)
	30–34	110 (17.8)
	> 34	62 (10)
Education	No formal education	61 (9.9)
	Primary school (grades 1–8)	168 (27.2)
	Secondary school (grades 9–12)	199 (32.3)
	Certificate or diploma	133 (21.6)
	First degree and above	56 (9.1)
Employment	Housewife	229 (37.1)
	Government employee	127 (20.6)
	Private employee	170 (27.6)
	Merchant	55 (8.9)
	Housemaid	36 (5.8)
Average family monthly income in ETB	< 3000 ETB	189 (30.6)
	3000–5999 ETB	273 (44.2)
	> 6000 ETB	155 (25.1)

### Obstetric factors

Out of 617 respondents, 268 (43.4%) were in the second trimester. Most of the pregnant women were multi-gravida (363/617, 58.8%) and were nulliparous (296/617; 48%). At the time of interview, 273 (44.2%) of pregnant women were on the first ANC visit (Table 2).

**Table 2:** Obstetric factors of pregnant women, Addis Ababa, 2017 (*n* = 617)

Variables		*n* (%)
Gestational period	First trimester	92 (14.9)
	Second trimester	268 (43.4)
	Third trimester	257 (41.7)
Previous pregnancy and delivery related problems	Yes	139 (22.5)
	No	478 (77.5)
Gravidity	Primigravida (1)	254 (41.2)
	Multigravida (> 1)	363 (58.8)
Parity	Nulliparous (0)	296 (48)
	Primiparous (1)	191 (31)
	Multiparous (> 1)	130 (21)
ANC Visit	First visit	273 (44.2)
	Second visit	186 (30.1)
	Third visit	99 (16)
	Four and above visit	59 (9.6)

**Fig. 1:**
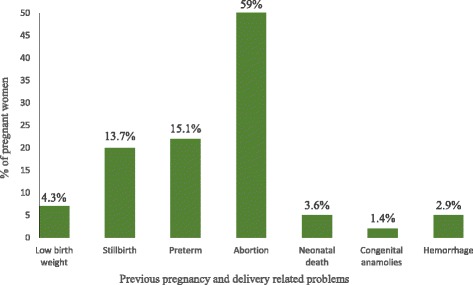
Previous pregnancy- and delivery-related problems encountered among pregnant women attending antenatal care in government health centers, Addis Ababa, 2017 (*n* = 139)

One hundred and thirty nine pregnant women (139/617; 22.5%) had encountered previous pregnancy- and delivery-related problems of which 82 (82/139; 59%) had encountered abortion (Fig. 1).

### Maternal health education and counseling

Four hundred and eighty-nine (79.3%) of the pregnant women attended maternal health education provided by the health centers during the antenatal care follow-up. More than half (338/617, 54.8%) of them had received an education on risk of self-medication, and 341 (55.3%) of them had received counseling services about risk of self-medication.

**Fig. 2:**
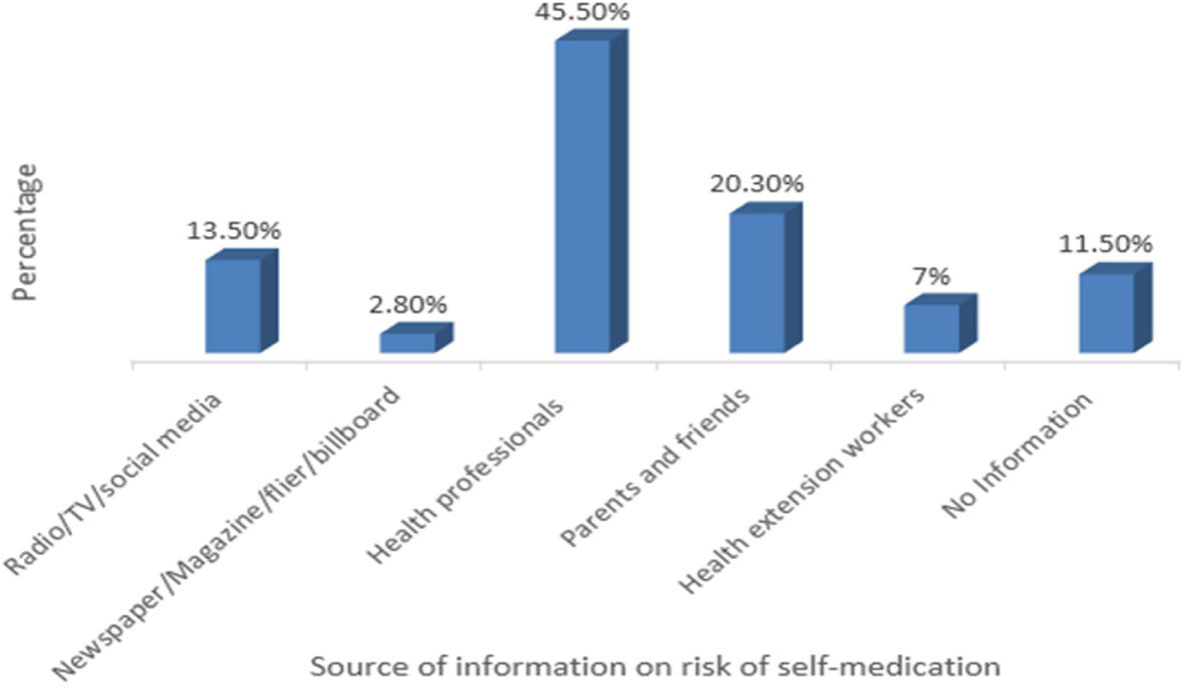
Source of information on risk self-medication during pregnancy, 2017 (*n* = 617)

Regarding the source of information about the risks of self-medication, majority (281/617, 45.5%) of the pregnant women obtained information from health professionals and 71 (11.5%) never obtained information from any sources (Fig. 2).

### Self-medication practice during pregnancy

The study participants used both modern and herbal medicines during the current pregnancy. Among the 617 pregnant women, 296 (48%) of them had previous self-medication experience and 164 (26.6%) practiced self-medication during the current pregnancy. From these who used medicines by themselves, 112 (18.2%) of them reported use of at least one modern medication while 67 (10.9%) of them took at least one herbal medicine. Fifteen (2.43%) participants took both modern and herbal medicines.

Among the 112 pregnant women who took modern medicines, the major reasons reported for self-medication were easy access to medicines without prescription from pharmacies or drug shops (80/112; 71.4%), thought that their disease was not serious (61/112; 54.5%) and said to save time (30/112; 26.8%). The sources of modern medicines for self-medication were pharmacies or drug shops (86/112, 76.8%), left-over medicines (21/112, 18.8%), and shared with family, friends or neighbors (11/112, 9.8%). Majority, 72 (64.3%) of the pregnant women decided by themselves to choose the modern medicines whereas 33 (29.5%) of the decision was made by their husband, family or neighbors (Table 3).

**Table 3:** Reasons for use, source of modern medication, and recommendation for self-medication of the pregnant women, Addis Ababa, 2017 (*n* = 112)

Variables		*n* (%)
Reasons for self-medication*	Time saving	30 (26.8)
	Obtaining medicines easily	80 (71.4)
	Disease not serious	61 (54.5)
	Self-medication is cheaper	20 (17.9)
	Previous medication experience	23 (20.5)
	Being embarrassed to tell about the disease	2 (1.8)
	High cost of visiting doctors and health service	5 (4.5)
	Poor health service provision	1 (0.9)
	Poor ethics of health professionals	1 (0.9)
	Long waiting time for health services	22 (19.6)
Source of modern medication*	Sharing with family, friends or neighbors	11 (9.8)
	Pharmacy or drug store	86 (76.8)
	Health facilities	1 (0.9)
	Left-over medicines	21 (18.8)
Recommended for self-medication by*	My Self	72 (64.3)
	My husband, family or neighbors	33 (29.5)
	Pharmacist or druggist	8 (7.14)
	Nurse	3 (2.7)

*More than one choice can be made by a pregnant woman

Among the 67 pregnant women who used herbal medicines, the reasons reported for self-medication were easily available herbal medicines (46/67; 68.7%), the disease was not serious (35/67; 52.2%), and believing that herbal medicines carry less risk than modern medicines (17/67; 25.4%). The sources of herbal medicines were homemade remedies (33, 49.3%), neighbors (23, 34.3%), and market or shops (7, 10.4%). The suggestions for herbal medicine use were made by the husband, family, or neighbors (35, 52.2%) and the pregnant women themselves (28, 41.8%) (Table 4).

**Table 4:** Reasons for use, source of herbal medication, and recommendation for herbal medication of the pregnant women, Addis Ababa, 2017 (*n* = 67)

Variables		*n* (%)*
Reasons for herbal medication*	Time saving	9 (13.4)
	Easily available herbal medicines	46 (68.7)
	Disease not serious	35 (52.2)
	Less risk of herbal medicines than modern medicines	17 (25.4)
	High cost of visiting health professionals and health service	3 (4.5)
	Long waiting time for health services	4 (6)
Source of herbal medications*	Family	5 (7.5)
	Homemade remedies	33 (49.3)
	Neighbors	23 (34.3)
	Market or shop	7 (10.4)
	Traditional healer	1 (1.5)
Recommended for herbal medication by*	My Self	28 (41.8)
	My husband, family or neighbors	35 (52.2)
	Traditional healer	2 (3)
	Traditional birth attendant	5 (7.5)

*More than one choice can made by a pregnant woman

In the present study, paracetamol (55/112; 49.1%) and amoxicillin (26/112; 23.2%) were the most commonly used medications for self-medication during pregnancy. Concerning the therapeutic category of the medicines, 76 (67.86%) were analgesics, 37 (33%) were antibiotics, and 17 (15.2%) were anthelmintics. As per US FDA pregnancy risk classification, 91 (81.25%) of the modern medicines used by the pregnant women were from category B; 24 (21.43%) were from category C; 19 (16.96%) were from category D; and 1 (0.89%) was from category X (Table 5).

**Table 5:** Modern medicines used for self-medication by pregnant women by trimester and US FDA pregnancy risk classification, Addis Ababa, 2017 (*n* = 112)

Medicines used	*n* (%)*	Trimester, *n*			US FDA risk category
		1st	2nd	3rd	
Paracetamol	55 (49.1)	27	22	6	B
Amoxicillin	26 (23.2)	12	14		B
Tinidazole	5 (4.5)	2	2	1	C
Metronidazole	4 (3.6)	1	2	1	B
Ibuprofen	6 (5.4)	2	4		C
Albendazole	6 (5.4)	4	2		C
Panadol	7 (6.3)	3	4		D
Diclofenac	5 (4.5)	1	3	1	C
Mebendazole	2 (1.8)	1	1		C
Augmentin (amoxicillin + clavulanate)	1(0.9)			1	B
Azithromycin	1 (0.9)		1		B
Tetracycline	3 (2.7)		3		D
Magnesium trisilicate	2 (1.8)	1	1		B
Ciprofloxacin	4 (3.6)	1	3		D
Aspirin	3 (2.7)	1	1	1	D
Bactrim (sulfamethoxazole + trimethoprim)	2 (1.8)	2			D
Ampicillin	2 (1.8)		2		B
Depo-provera (medroxyprogesterone)	1 (0.9)	1			X
Unknown medicines	3 (2.7)	2	1		

*More than one medicine could be used by a pregnant woman

In this study, basil (47/67; 70.2%), rue (15/67; 22.4%), garlic (7/67; 10.5%), and ginger (5/67; 7.5%) were the commonly used herbal medicines for self-medication by the pregnant women (Table 6).

**Table 6:** Herbal medicines used for self-medication by the pregnant women by trimester, Addis Ababa, 2017 (*n* = 67)

Herbal medicines	*n* (%)^a^	Trimester, *n*		
		1st	2nd	3rd
*Zingiber officinale* (ginger)	5 (7.5%)	2	2	1
*Allium sativum* (garlic)	7 (10.5%)	6	1	
*Ocimum lamiifolium* (basil or damakasie)	47 (70.2%)	26	18	3
*Hagenia abyssinica* (koso)	1 (1.5%)	1		
*Taverniera abyssinica* (emergency herb)	1 (1.5%)	1		
*Ruta chalepensis* (rue or tena adam)	15 (22.4%)	10	3	2
*Lepidium sativum* (peppergrass or feto)	4 (6%)	2	2	
*Coffea arabica* linn (altet)	2 (3%)	1	1	
*Eucalyptus globulus* (eucalyptus leaf)	1 (1.5%)		1	
*Brassica nigra* (mustard seed)	1 (1.5%)		1	
Others*	3 (4.5%)	2	1	

Others*, dign, shikukonina and roots

^a^More than one herbal medicine could be used by a pregnant woman

### Knowledge about risk of self-medication

From the total study participants, more than half (327/617, 53%) of the pregnant women had a good knowledge about risk of self-medication during pregnancy, and 290 (47%) had poor knowledge.

### Factors associated with self-medication practice during pregnancy

In univariate analysis, age, employment status, average monthly family income, gestational period, maternal education, education on self-medication, counseling, previous pregnancy- and delivery-related problems, previous medication use, and knowledge about risk of self-medication were significantly associated with self-medication practice. However, in multivariable logistic regression analysis, only gestational period, previous pregnancy- and delivery-related problems, previous medication use, education on self-medication, and knowledge about risk of self-medication were found to be significantly associated with self-medication practice during the current pregnancy.

In this study, the gestational period was significantly associated with self-medication practice. The odds of self-medication practice among women in their second trimester of pregnancy were 0.63 times the odds of those in their third trimester (AOR = 0.63, 95% CI 0.406–0.984).

Previous medication use was strongly associated with self-medication practice. Pregnant women who used medications before the current pregnancy were about four times more likely to practice self-medication during the current pregnancy as compared to those who did not use medications (AOR = 4.20, 95% CI 2.695–6.534). In addition, pregnant women who encountered previous pregnancy- and delivery-related problems were 1.7 times more likely to practice self-medication than those who did not encounter (AOR = 1.71, 95% CI 1.060–2.761).

Education on self-medication was also strongly associated with self-medication practice. The odds of self-medication practice among pregnant women who were educated about self-medication consequences were 0.36 times the odds of those who were not (AOR = 0.36, 95% CI 0.209–0.617). Moreover, the odds of self-medication practice among pregnant women who had good knowledge about risk of self-medication practice were 0.64 times the odds of those who had poor knowledge (AOR = 0.64, 95% CI 0.419–0.973) (Table 7).

**Table 7:** Univariate and multivariable analysis of factors associated with self-medication practice among pregnant women in Addis Ababa, 2017 (*n* = 617)

Variables	Self-medication practice		COR (95% CI)	AOR (95% CI)	*p* value
	Yes	No			
Age in years					
18–24	46	148	0.64 (0.381–1.072)	0.81 (0.446–1.475)	0.492
25–29	60	191	0.65 (0.395–1.057)	0.72 (0.419–1.246)	0.242
30–34	36	74	1.00	1.00	
> 34	22	40	1.13 (0.587–2.177)	1.002 (0.474–2.118)	0.996
Employment					
Housewife	55	174	0.69 (0.425–1.113)	0.82 (0.464–1.448)	0.494
Government employee	40	87	1.00	1.00	
Private employee	47	123	0.83 (0.503–1.375)	0.91 (0.514–1.622)	0.756
Merchant	13	42	0.67 (0.326–1.391)	0.68 (0.304–1.513)	0.343
Others*	9	27	0.73 (0.312–1.683)	0.61 (0.229–1.627)	0.324
Average monthly family income					
< 3000 ETB	54	135	1.00	1.00	
3000–5999 ETB	63	210	0.75 (0.491–1.145)	0.83 (0.517–1.334)	0.442
> 6000 ETB	47	108	1.09 (0.683–1.733)	1.47 (0.831–2.598)	0.185
Gestational Period					
First trimester	21	71	0.67 (0.383–1.160)	0.61 (0.326–1.128)	0.114
Second trimester	64	204	0.71 (0.480–1.040)	0.63 (0.406–0.984)	0.042*
Third trimester	79	178	1.00	1.00	
Maternal Education					
Yes	115	374	0.50 (0.328–0.479)	1.08 (0.631–1.851)	0.777
No	49	79	1.00	1.00	
Education on self-medication					
Yes	56	282	0.31 (0.216–0.457)	0.36 (0.209–0.617)	***<*** 0.001*
No	108	171	1.00	1.00	
Counseling					
Yes	69	272	0.48 (0.336–0.693)	1.28 (0.706–2.321)	0.416
No	95	181	1.00	1.00	
Previous medication					
Yes	121	175	4.47 (3.008–6.642)	4.20 (2.695–6.534)	< 0.001*
No	43	278	1.00	1.00	
Previous pregnancy- and delivery-related problems					
Yes	51	88	1.87 (1.249–2.805)	1.71 (1.06–2.76)	0.028*
No	113	365	1.00	1.00	
Knowledge					
Poor knowledge	96	194	1.00	1.00	
Good knowledge	68	259	0.53 (0.369–0.762)	0.64 (0.419–0.973)	0.037*

*AOR* adjusted odds ratio, *COR* crude odds ratio, *CI* confidence interval

*Statistically significant (*p* < 0.05)

### Qualitative findings

A total of nine in-depth interviews were conducted with the key informants. The key informants were two pharmacists, two health officers, three midwives, and two nurses all with a bachelor degree and assumed roles as directors, team leaders, ANC program coordinators, or ANC case team leaders. Their age ranges from 25 to 37 years with 2 to 10 years of work experiences in various organizations.

The key informants were asked about the overall situations, guidance, and policies of self-medication practice among pregnant women. Key themes emerged from the analysis were (i) self-medication practices; (ii) regulatory enforcement; (iii) education and counseling on self-medication; (iv) strategies, guidelines, and forms related; and (v) reasons for self-medication. Analysis of the qualitative data from interviews with key informants is presented in detail as follows.

### Self-medication practice

The participants thought that pregnant women were cautious not to use medicines without consulting health professionals. The majority of the participants believed that the trends of using medicines by the pregnant women were decreasing. However, they mentioned that since medicine retail outlets sale medicines without prescription, the pregnant women might use medicines without prescription. This was supported by one of the participant that:

“….. Although the pregnant women feel that self-medications are risky for them and the fetus, they [the pregnant] believe the recommendations from families or friends and this has encouraged them to use medicines by themselves” (SMP01).

Another participant also reinforced the ease of obtaining medicines without prescription from pharmacies or drug shops as follows:

“……. If a pregnant woman asks for a medicine, including antibiotics from pharmacies or drug shops, she can obtain the medicine easily. I feel that the dispensing practice is not as per the regulatory requirements and the regulatory body has not been controlling selling of prescription medicines without prescription” (SMP03).

One of the participants, however, was uncertain on whether self-medication practice among the pregnant women is increasing or decreasing. She pointed out that:

“………The trend of using medicines without prescription among pregnant women seems to decrease, but there is an increase in dispensing of medicines including antibiotics without prescription by pharmacies or drug shops. Hence, the cumulative effect of medicine sale to pregnant women without prescription might be higher or lower” (SMP02).

### Reasons for self-medication practice

The participants argued that there are situations where pregnant women practice self-medication and different reasons were identified for such practices. Advice from families or friends or neighbors, inconsistent maternal education including risk of self-medication, poor awareness on risk of self-medication, time saving, feeling that the disease is minor, lack of attention and priorities of program designers, communication gap between pregnant women and health care providers, impairment of continuous assessment of women during pregnancy for self-medication, and weak regulatory enforcement were the major reasons for self-medication practice.

### Weak regulatory enforcements

Weak regulation and enforcement on restricting nonprescription medicine sale to pregnant women was mentioned as one of the reasons for self-medication. One of the respondents indicated that unethical dispensing of medicines without prescription may not be due to the lack of knowledge about its consequences for pregnant women but it is due to lack of strong regulatory enforcements. One of the participants mentioned that:

“….These times it is easy to buy any medicine without a prescription from the retail outlets. This is due to the weak regulatory enforcement and I think this weak regulatory system is the main reason for the sale of medicines without prescription” (SMP04).

The respondents suggested the need for enforcing the regulation so as to protect the pregnant women from access to prescription medicines for self-medication. Moreover, the respondents said that even though the health professionals did not have a knowledge gap about the risk of self-medication, they emphasized the need to continuously update on the extent and burden of self-medication. One of the participants supplemented that:

“……… Dispensers may know the consequence of self-medication. But, I don’t think they are well aware of its burden. Hence, they should be well oriented on the impact of self-medication during pregnancy” (SMP05).

### Strategies, guidelines, and forms or charts

The respondents stated that there is no written strategy or guidelines, including charts that articulate self-medication practice during pregnancy. As claimed by the participants, there is no guidance on self-medication. However, there is a strategy about maternal and reproductive health including antenatal care programs. But, this strategy does not address self-medication practices and its interventions. One of the respondents elaborated it in that the strategy fails to address risks of self-medication practices. They stated that it looks either the problem is not so well known or there is lack of priority and attention to the matter among policy makers and program designers.

Another important finding was that the ANC follow-up records missed to include a section for documenting self-medication which would have reminded ANC service providers to receive history and counseled pregnant women on taking caution on self-medication. One of the respondents highlighted as follows:

“……… The client screening chart used in the health center is not designed to have spaces where ANC providers write their concern of self-medication history” (SMP06).

The findings indicated that most of the screening activities for self-medication has been triggered when the pregnant women explained that she was sick and treated for her ailments. There is no procedure to ask the mother about self-medication and no guidance documents about risk of self-medication. This hinders recording of self-medication history of pregnant women during her follow-up.

### Education and counseling

The majority of the respondents reported that maternal education, including education on self-medication is expected to be given to the pregnant women. However, this has been done inconsistently. This is because the education and counseling packages focus on danger signs and symptoms of pregnancy. It does not include risk of self-medication practice as a program. This results in poor awareness of the pregnant women on risk of self-medication. If a pregnant woman found a medicine effective based on her previous medication history, she will use the medicine during the current pregnancy. This was augmented by one of the respondents as follows:

“…… Pregnant women may request a medicine from a pharmacy or drug shop based on the effectiveness of their previous treatment experiences. They confidently request for a medicine if they feel the symptom of a disease seems the same as before. This is due to the knowledge gap on risks of using medicines to the fetus as well as her health” (SMP04).

The limited education and counseling of the pregnant women on risk of self-medication depends on the awareness and in-service training of the ANC service providers and other health professionals. Some respondents agreed that there were trainings on maternal and child health components to health professionals but the concept of risk of self-medication was missed. They also echoed that most of the providers at the health centers are junior staff and are less experienced. This exacerbated the limited focus to risk of self-medication. One of the respondents alluded that:

“……… I have never taken a training on medicine use risks including risks of self-medication during pregnancy. I have also never encountered national guidance documents on risk of self-medication which I may look up in my practice” (SMP07).

The other issue raised by one of the respondents was about the need for standardized self-medication related guidance. This was highlighted as shown below:

“……..National guidance documents on risk of self-medication are necessary to protect the health of the pregnant women and fetus. Moreover, continuous follow up and in-service trainings are important for the betterment of the ANC services and health of the pregnant women” (SMP06).

## Discussion

The present study assessed self-medication practice and associated factors among pregnant women in government health centers in Addis Ababa, Ethiopia. This study found out that a considerable proportion of pregnant women practiced self-medication with at least one modern and/or herbal medicine during the current pregnancy without consultation of health professionals. These findings were almost similar compared to studies conducted in the Democratic Republic of Congo, Nigeria, and south west part of Ethiopia [6, 16, 29]. More than a quarter (26.6%) of pregnant women practiced self-medication during their current pregnancy. The prevalence of self-medication practice in our study was higher when compared to reports in Iran, Pakistan, and Brazil [34–36] and lower than reports in Peru, Portugal, the Netherlands, Brazil, and Iran [18, 34, 38–41]. The possible reasons for the difference might be due to differences in study methods, health care settings, and restriction policies of dispensing practices.

The reasons why pregnant women practiced self-medication were easy access to medicines from pharmacies or drug shops without prescription followed by time saving, long waiting time for provision of health services, feeling that illness was not serious, and self-medication is cheaper. The easy access to medicines without prescription might be due to inadequate regulatory enforcements of dispensing practices and the imbalance between medicine trade and professional ethical standards among dispensers. This is supported by the qualitative findings, which showed that there is a gap in regulatory enforcements and lack of attention and priorities of policy makers and program designers on the burden of self-medication risks. This finding was similar to other studies conducted in Peru, Europe (Western, Northern and Eastern), North and South America and Australia, the Democratic Republic of Congo, and Ethiopia [6, 16, 37, 38].

Analgesics, antibiotics, and anthelmintics were the most commonly used classes of medicine for self-medication by the pregnant women, whereas paracetamol and amoxicillin were the most frequently used modern medicines. Studies from the Democratic Republic of Congo and south west part of Ethiopia had reported similar findings [6, 16]. Mainly, those medicines were obtained from pharmacies or drug shops. The reason for antibiotic medicines dispensing without prescription might be due to the weak enforcement of regulation and less strict dispensing policies. This may result in increased treatment costs, adverse effects, and the emergence of antimicrobial resistance.

Left-over medicines are other sources of modern medicines for self-medication by the pregnant women. This was found to be consistent with a study done in Peru [38]. As left-over medicines might be expired, inadequate in dosage, or contraindicated during pregnancy, such kind of practice should be discouraged. Sharing of medications is another source of modern medicines which was similar to a study done in Bahirdar, Ethiopia [27]. This indicated that families and friends have an influence on self-medication. This might be due to poor awareness about risk of self-medication, risk of sharing, and using left-over medicines.

The most worrisome issue in our findings is that pregnant women used herbal medicines which may be risky for the fetus. For instance, *Zingiber officinale* is known for its abortifacient, emmenagogue, and mutagenic effects [27, 48]. The reasons for self-medication of herbal medicines were easily available herbal medicines, feeling that the illness was not serious, perception of herbal medicines is less risk than modern medicines, and time saving. This finding was supported by other studies in Nigeria and Sudan [29, 32]. Furthermore, the sources of the herbal medications reported by the pregnant women were homemade remedies and neighbors. This could be due to family influences and sharing as a socio-cultural support for using and suggesting herbal medicines.

The findings of this study revealed that gestational period was significantly associated with self-medication practice. This is similar to a study conducted in Southern Iran [14]. The odds of self-medication practice among women in their second trimester of pregnancy were 0.63 times the odds of those in their third trimester. This might be due to the fact that initial discomforts associated with new pregnancy and most symptoms of pregnancy-related illness are during the first stage of pregnancy. Moreover, pregnant women might be less careful in the last trimesters due to the assumptions of the maturity of the fetus.

Pregnant women who had a history of self-medication practice before the current pregnancy were about fourfold more likely to practice self-medication during the current pregnancy as compared to those who had not. This might be due to the presence of chronic diseases, previous exposure to medicines, pregnancy- and delivery-related problems, and poor awareness on risk of self-medications. Another possible reason could be that commonly used medicines outside pregnancy might create the impression that over-the-counter medicines are safe to use during pregnancy. This study was in congruence with studies done in Iran, the Democratic Republic of Congo, Nigeria, and Jimma, Ethiopia [6, 14, 16, 29].

Pregnant women who encountered pregnancy- and delivery-related problems before were nearly twice more likely to practice self-medication than those who did not encounter. The possible reasons might be because the previous experience in using medication to treat problems related to pregnancy and delivery adverse outcomes and considered the medicines used were safe. These findings are almost similar to studies conducted in the Democratic Republic of Congo [6].

Pregnant women who were not educated about self-medication consequences were more prone to self-medication practices. The odds of self-medication practice among pregnant women who were educated about self-medication consequences during pregnancy were 0.36 times the odds of those who were not educated. This was augmented by the qualitative findings. Pregnant women who did not attend their regular ANC follow-up and maternal education on the consequences of self-medication had practiced self-medication. Lack of awareness on potential risks of self-medication and a communication gap between pregnant women and health care providers might be the possible reasons. This was complemented with other studies conducted in India and Sudan [22, 32, 34].

Moreover, the odds of self-medication practice among pregnant women who had good knowledge about risk of self-medication practice were 0.64 times the odds of those who had poor knowledge. The possible reasons could be poor awareness on risk of self-medications during pregnancy and the pregnant women believed the advices of their families, relatives or friends. The study showed, for example, some pregnant women believed that self-medication during pregnancy saved lives of many unborn babies than harm; it is better for the fetus that the mother uses medicines by herself and get well than to have an untreated illness during pregnancy; self-medication during pregnancy do more good than harm; pregnant women should not consult health professionals before taking herbal medicines; and herbal medicines use during pregnancy are safer than modern medicines. This finding was comparable to other studies done in Saudi, Western India, and Sudan [22, 30, 32].

Self-medication practice is one of the most common public health concerns during pregnancy. Unless necessary care is provided by responsible health professionals, it may lead to high risk, including maternal and neonatal mortality and morbidity [3, 27, 29]. In this study, more than one third (40%) of the pregnant women had self-medicated with medicines from category C, D, and X which are thought to cause possible fetal harm and high risk to the fetus and women. This indicated that some pregnant women were potentially at higher risk. For instance, aspirin and ibuprofen cause premature closure of ductus arteriosus during the third trimester, miscarriage, cardiac malformation, fetal renal impairment, pulmonary hypertension, and delayed onset of labor and prolongation of bleeding time in mother; paracetamol, the commonest non-steroidal anti-inflammatory drugs (NSAIDs), may also cause a risk of attention deficit hyperactivity in babies and reduced implantation sites at any time of pregnancy. Antihelmentics such as albendazole, mebendazole, metronidazole, and tinidazole cause incidence of fetal mortality, fetotoxicity, embryotoxicity, and skeletal malformations.

Furthermore, the use of medroxyprogesterone causes teratogenicity, fetotoxicity, fetal malformation, low birth weight, and neonatal death, and the use of tetracycline causes permanent discoloration of teeth, enamel hyperplasia, and impaired fetal skeletal growth [11].

Pregnant women should not only be cautious about modern medicines during pregnancy, they should also be cautious about commonly used herbal medicines. Since those herbs are understudied specially in pregnancy and their compositions are not well known, some of them cause serious risks [49]. For example, *Ocimum lamiifolium* (basil) contains eugenol and estragol with anticonvulsant and sedative antispasmodic effects; *Allium sativum* (garlic) has uterine stimulant effect; and *Ruta chalepensis* (rue) contains furanocumarins and is embryo toxic causing implantation failure and abortion. *Lepidium sativum* (peppergrass) contains sodium benzylisothiocyanate which induced low fetal and placental weights [27, 29, 48, 50, 51].

### Strengths and limitations

The strengths of the study were that the quantitative data were triangulated with qualitative findings. Furthermore, the study considered factors such as knowledge, education on self-medication, counseling, previous pregnancy- and delivery-related problems, and policy- and program-related matters such as screening mechanisms for self-medication practice and adequacy of regulatory enforcements. The study had some limitations as well. Recall bias among pregnant women due to the fact that they were expected to recall information from their past experiences might affect the study findings. The other limitation of the study was that pregnant women might be confused or embarrassed to report the use of medicines during data collection since the study was institutional. In addition, the study might had response bias among key informants.

## Conclusions

A considerable proportion of pregnant women attending antenatal care in government health centers in Addis Ababa practiced self-medication with at least one modern and/or herbal medicines. Gestational period; previous medication use; education on self-medication; previous pregnancy- and delivery-related problems; and knowledge about risk of self-medication were significantly associated with self-medication practice during the current pregnancy.

There is lack of attention among policy makers and program designers on addressing the risk of self-medication. There were no guidelines and recommendations on self-medication during pregnancy that ANC providers can use and there was no screening mechanisms for history of medication. There was also weak regulation enforcement on medicine retail outlets as they were good sources of medicines for self-medication. In addition, the pregnant women have limited awareness on self-medication, and they believe the advices of their families and friends.

The ANC follow-up records do not trigger health care providers to write and follow histories of self-medication of the pregnant women. For this reason, necessary measures at all levels must be taken to reduce the risks of self-medication during pregnancy.

Therefore, it is important to aware the pregnant women with risks of self-medication and train health care providers on how to help pregnant women stay safe from self-medication. Moreover, concerted efforts need to be exerted to strengthen regulatory enforcements and routinely screen pregnant women. It is also important to formulate and/or revise maternal and child health including ANC program-related strategies, guidelines, and ANC charts or forms to address risk of self-medication practices.

We recommend further research to assess the consequences of self-medication practice on pregnancy outcomes and community-based studies to identify factors for self-medication practice.

## Abbreviations

AAHB: Addis Ababa Health Bureau
ANC: Antenatal Care
ETB: Ethiopian Birr
SDG: Sustainable Development Goal
SPSS: Statistical Product and Service Solutions
